# Implementation and implications for polygenic risk scores in healthcare

**DOI:** 10.1186/s40246-021-00339-y

**Published:** 2021-07-20

**Authors:** John L. Slunecka, Matthijs D. van der Zee, Jeffrey J. Beck, Brandon N. Johnson, Casey T. Finnicum, René Pool, Jouke-Jan Hottenga, Eco J. C. de Geus, Erik A. Ehli

**Affiliations:** 1 Avera Institute for Human Genetics, Avera McKennan & University Health Center, Sioux Falls, SD USA; 2 Department of Biological Psychology, Netherlands Twin Register, Vrije Universiteit Amsterdam, Amsterdam, Netherlands

**Keywords:** Polygenic risk score, PRS, Clinical genetics, Genetic risk, Risk stratification, Public health

## Abstract

Increasing amounts of genetic data have led to the development of polygenic risk scores (PRSs) for a variety of diseases. These scores, built from the summary statistics of genome-wide association studies (GWASs), are able to stratify individuals based on their genetic risk of developing various common diseases and could potentially be used to optimize the use of screening and preventative treatments and improve personalized care for patients. Many challenges are yet to be overcome, including PRS validation, healthcare professional and patient education, and healthcare systems integration. Ethical challenges are also present in how this information is used and the current lack of diverse populations with PRSs available. In this review, we discuss the topics above and cover the nature of PRSs, visualization schemes, and how PRSs can be improved. With these tools on the horizon for multiple diseases, scientists, clinicians, health systems, regulatory bodies, and the public should discuss the uses, benefits, and potential risks of PRSs.

## Introduction

Determination of risk has been central to disease assessment and prevention for decades within the medical field [63]. Currently, the focus on disease prevention and mitigation has become a major focus of healthcare institutions in order to reduce the strain of human disease on public health systems. By enhancing the capacity for healthcare workers to intervene early with patients, guiding them towards healthier lifestyles and better personal choices, the hope is to prevent the worse effects of disease later in life and more effectively use healthcare resources [63].

At the beginning of the twenty-first century, nearing the completion of the Human Genome Project, there was hope that acquiring the human genetic blueprint would allow us to understand disease and determine those at risk quickly through genetic testing [11]. However, these ideas were far too optimistic. Instead of simple connections between a specific gene or set of genes and their associated disease, we found a complex web of interactions throughout the genetic code involving hundreds to millions of single nucleotide polymorphisms (SNPs) [52], especially among more common and complex diseases. Such diseases have a polygenic underpinning involving thousands of genetic variants that each has only a small effect on the disease process. This reality fundamentally limits the ability of single or multi-candidate gene testing for use in risk-assessment and diagnosis of common diseases [35].

**Fig. 1:**
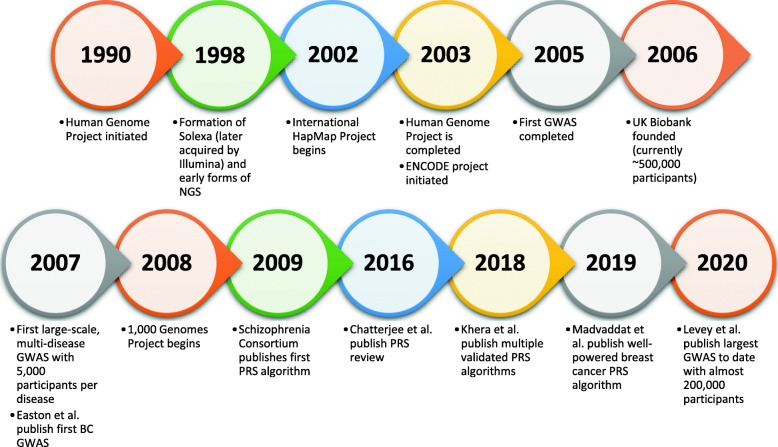
Timeline of major events in genomics since the start of the Human Genome Project until today. Note the acceleration of advancements and increasing scale of studies

The discovery of multi-gene diseases forced researchers to backtrack on their original claims and hopes for genetic testing, putting the idea of genetic diagnosis and risk assessment [11] on the backburner. From this effort arose a modern expansion in the field of quantitative genetics and led to the advent of the genome-wide association study (GWAS), with the first completed in 2005 with only 24 cases and controls [24]. The first large-scale GWAS occurred in 2007 with ~ 2000 cases for 7 diseases and ~ 3000 shared controls [14]. Over time, more and more GWASs were performed with ever increasing numbers of subjects, some even over 1 million subjects [32, 42]. These advances, among others, have led to increased study power and the ability to capture more genetic variants associated with a given trait such as diabetes [66], cardiovascular disease (CVD) [36], anxiety [43], and even depression (see Fig. 1) [28].

In 2009, the International Schizophrenia Consortium demonstrated, for the first time, the ability to use a multi-gene profile to categorize and stratify individuals based on their likelihood of developing schizophrenia [31]. This was the first example of a polygenic risk score (PRS) model being used and the researchers were able to demonstrate the power of PRSs for disease stratification (International Schizophrenia et al. 2009 [63]). The study had large implications for the future of genetic testing, public health, and clinical care. Chatterjee et al. later published a seminal review on PRS development and evaluation, emphasizing the need for absolute risk estimates to be the focus of future PRS development [7]. Researchers then continued this effort for more diseases [39] and even began to integrate clinical risk factors with PRSs to increase risk stratification accuracy [41], acknowledging the inherent and intricate gene-environment interactions in complex human disease. Most notable is the study published by Khera et al. in 2018 which showcased the use of PRSs for coronary artery disease (CAD), breast cancer, atrial fibrillation, inflammatory bowel disease, and type II diabetes [35].

The hopes for genetic testing being useful and informative in the clinic have been revitalized. Advances in PRS development provide new and reliable possibilities for determining an individual’s disease risk which, in practice, could encourage preventative screening or treatment, furthering the possibility of avoiding the disease altogether. In addition, a framework to build upon was established and the integration of clinical/environmental risks is continuing. In many ways, PRSs hold true to the original promise of genetic testing for patients and the public. This revelation opened the doors for numerous consortia and bioinformatics groups to assess their repositories and to analyze their databases for any information that they could glean, the more subjects the better. This marked a milestone in the applications of big data science in genetics and new efforts were put underway to collect ever more genetic data from individuals. Now, GWASs may not only be a preamble for future research into the genes and molecular mechanisms of disease, but a clinical tool for disease risk assessment.

The past years have shown an explosive growth of the scientific application of PRSs which fall in five broad classes: verification of genome-wide associations, establishing genetic correlation between traits, testing gene-by-environment interaction, stratification by genetic risk to establish causal effects that are independent of genetic confounding, and the establishment of ‘genetic nurture.’ To verify the predictive ability of SNPs identified by GWAS meta-analyses for the trait-of-interest, which is critical for their use in the clinic, PRSs are computed in out-of-sample cohorts and regressed on the trait to estimate the amount of variance explained by the PRS in the target population [35, 46]. The use of an independent out-of-sample cohort is mandatory because when the “target” sample is part of the “discovery” sample, the prediction is going to be inflated [64]. PRS is also a powerful tool to establish a shared genetic basis between apparently unrelated traits, as shown for example by the prediction of creativity by the PRS for schizophrenia and bipolar disorder [56]. PRSs, in sharp contrast to isolated candidate genes [16], further enable well-powered tests of gene-by-environment interaction which surprisingly falsified the hypothesized interaction between genetic susceptibility for depression and childhood trauma [54]. Stratification on PRS scores uniquely enables causal inference in observational studies that is corrected for genetic confounding. For example**,** Choi et al. [8, 9], combining a lifestyle survey on recreational physical activity with electronic health records on incident episodes of depression, showed that even individuals with high genetic vulnerability for depression could avoid new depressive episodes when they are sufficiently physically active [8]. Finally, using a separate PRS based on the transmitted and on the untransmitted alleles of the parents, we can separate the genetic and environmental components present in intergenerational transmission. This was, for example, used to show that the rearing environment provided by the parents to their offspring (genetic nurturing) played an important role in the child’s educational attainment, but did not affect the transmission of vulnerability for ADHD which was entirely genetic [17].

Whereas there is now ample demonstration of the use of PRS to advance science, their clinical applications are still rare. Here, we focus on the clinical implementation of PRSs and their utility both to the larger healthcare system and to individual health outcomes. We begin by briefly discussing how PRSs are calculated and the science behind them. We then evaluate their potential use in the clinic and the challenges inherent to their integration into healthcare at large, especially in training healthcare professionals and explaining the results to patients. Later, we discuss methods of visualization and communication of polygenic risk and the implications this information has for patients and their families. Finally, we discuss the ethical status of PRSs and the potential risks in using the technology, especially on underrepresented populations in genetic studies.

For clarity, we often discuss the use of PRSs as *scores* which will be the main tool that healthcare providers will use and discuss with patients. These scores are generated via algorithms which can contain tens to millions of SNPs depending on the disease/phenotype of interest. Throughout this review, we will focus on the scores themselves but will at times discuss the algorithms (i.e., PRS algorithms) and how they function in particular versus the scores (i.e., PRSs) that will be used in the clinic.

Given recent guidelines published by Khan et al. [34], we clarify that this work will focus almost exclusively on *genetic* ancestry (often referred to simply as “ancestry”) and not on race and ethnicity. While (genetic) ancestry, race, and ethnicity are often linked, they are not equivalent terms and we endeavor to be as specific as possible regarding the terminology used within this work. We do, however, discuss some of the ethical impacts regarding PRS implementation and therefore discuss its potential effects on racial and ethnic health disparities. Given that these are sociological terms and we are referring to social issues, we then use the terms “race” and “ethnicity” directly. 

## How PRSs are constructed

A PRS represents a calculated estimate of trait or disease liability according to an individual’s genetic profile in the context of relevant GWAS summary statistics. Specifically, a PRS is computed by summing the number of risk alleles (0, 1, or 2) that an individual possesses (target data), weighted by the risk allele effect sizes as determined by a GWAS on the phenotype of interest (discovery data). The effect size is expressed as the log (OR) for binary traits or slope of the linear regression between allele count and trait for continuous traits. Questions then arise as to the number of risk alleles important for defining the most accurate PRS. Studies have demonstrated that PRSs achieve greater predictive power when they include a large number of genetic variants, also known as SNPs, rather than limiting a PRS to only those SNPs that attain genome-wide significance in the associated GWAS [1, 31, 48].

Rigorous quality control measures must be implemented on both the discovery and target data to achieve validity and power in the PRS calculation. Particular care must be taken during the quality control stages because small errors can become magnified during the aggregation of SNP effects in PRS computation. Researchers and clinicians alike should familiarize themselves with and become knowledgeable of standard quality control steps (see this tutorial [9]) in PRS calculation as these analyses can have severe outcomes on the accuracy of the PRS.

**Fig 2:**
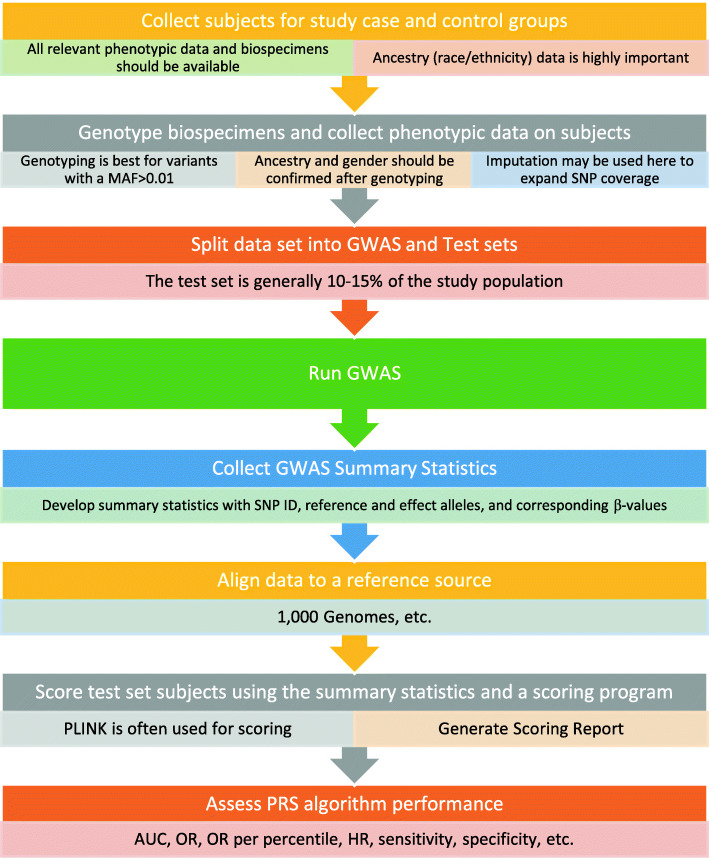
General scheme and important considerations for PRS development. This figure begins with collection of study participants and ends with assessment of PRS algorithm performance

Various strategies have been developed to select the genetic variants that are used in PRSs [10, 65]. When calculating a PRS, it is important to consider factors that contribute to violating the assumptions of normality. That is, the PRS is the sum of independent variables (i.e., SNPs) with identical distributions; thus, the PRS should be approximately a normal distribution even when the predictive power is low. A PRS with a non-normal distribution likely results from inclusion of many correlated SNPs (due to linkage disequilibrium) or derivation and application of the score from heterogeneous populations (SNPs with markedly different allele frequencies and genotype distributions). Therefore, evaluation of PRS distributions may elucidate errors in PRS calculation or issues that persist due to not properly addressed population stratification existing in the target sample. When the PRS is normally distributed, Z-scores can be calculated, helping to improve score interpretation and increase the comparability across traits. A general schematic of this process is represented in Fig. 2.

## PRS in the health care system

The potential for PRSs in the clinic is far reaching for multiple levels within the healthcare system. Specifically, the potential clinical implications of PRSs can be subdivided into two major areas: (1) public health and (2) individual and clinical decision-making.

### PRS’s influence on public health

Within the domain of public health, prevention is one of the largest initiatives to help maintain a healthy population and to reduce the morbidity, mortality, and economic costs of disease on society. Prevention is generally broken down into three categories: primary, secondary, and tertiary. Primary prevention involves disease reduction through the use of information, healthy behaviors, and regular health maintenance to prevent a disease, such as avoiding smoking for lung cancer. Secondary prevention involves testing patients for early detection (cholesterol screening, etc.), commonly referred to simply as screening. Screening is used routinely in the clinic, from various procedures, such as mammograms for breast cancer and colonoscopies for colon cancer, questionnaires, like those for clinical depression and anxiety, to blood tests, such as cholesterol testing for CAD. When applied properly, screening is used to discover a disease early in its pathophysiology, enabling early intervention with the goal of either curing the individual of the disease or preventing the disease from progressing. By doing so, individuals who need more invasive, and potentially harmful diagnostic tests, can be found while the rest of the population is not put at risk. Finally, tertiary prevention involves medications or procedures designed to intervene and reduce adverse outcomes in patients [38].

PRSs likely fit into this system as a hybrid of primary and secondary prevention, functioning as an early secondary prevention that may be able to influence primary prevention strategies. While a DNA sample from a patient would be required for evaluation, PRS analysis on an individual would ideally occur long before an individual has any signs or symptoms of the disease in question. Instead, a PRS for a number of diseases would be generated early on, giving clinicians and other healthcare workers a jump-start in initiating conversations regarding healthy habits, access to health programs, and other preventative measures (medications, further testing, etc.) [63]. This may have a profound effect on the prevention of diseases, intervening even before exposure to a potentially harmful environmental risk factor, and greatly reducing the likelihood of developing a condition later in life.

Moreover, PRS-based information could help to improve healthcare resource optimization to reduce costs and justify, if applicable, increased spending on certain diseases with high genetic burden in larger populations, such as a nation or state. This information could be used in combination with socioeconomic and existing healthcare data to argue for increased public spending and healthcare outreach efforts. Public health awareness campaigns and increases in funding have been shown to increase overall health outcomes in the past and could be further justified with PRS information on a large enough scale. Outcome changes could be monitored using deidentified/anonymized data to observe the effects of enacted public policy changes on genetically at-risk (sub)populations, representing an improvement in overall well-being and health of communities. Even greater would be the increased knowledge that such a scenario would offer for increasing the accuracy of PRSs for individuals given the increase in deidentified data available for research barring subject informed consent and internal review board approval.

### Individual and clinical decision making:

The ability to stratify on the population has great benefits to society, but PRSs have an even greater potential for individual patients. By having a PRS, a patient will have a quantitative measure of their individual risk for developing a given disease. In consultation with their physician, a specific and personalized plan can be developed. Most patients will not require special care, but for the intermediate and high-risk groups, different or increased screening may be necessary [31, 63]. For example, in the USA, women ages 50–74 are recommended to have a mammogram every 2 years if they are at average risk [61]. But for those women who are at increased risk, this mammogram interval may be decreased to one every year or breast magnetic resonance imaging (or MRI) may be warranted to reduce lifetime radiation exposure in high-risk women [48]. For the most severe cases, radical double mastectomy and total hysterectomy may be warranted [41, 48].

As a local physician, PRSs function as a tool to better understand a patient than family history can offer alone [63]. These scores provide a quantifiable and objective measure of genetic risk compared to the subjective nature and potential recall bias of family history [3, 59] and self-reported lifestyle risk factors [53, 57]. Patients, through no fault of their own, often do not know the exact history of their parents or grandparents; vital information for assessing risks involving diabetes, cancer, and CAD. PRSs offer another metric for measuring inherited risk [26].

Given genome-wide SNP genotyping, assessing genetic risk should only require a single DNA sample for assessment. While whole genome sequencing may become universally available in time, methods such as array-based genotyping paired with SNP imputation can be used to gather genome-wide information for individuals, such as the Illumina Global Screening Array [4]. This digitized genome-wide information can then be used to score a patient for any number of diseases with available PRSs. Given that the genome does not change, this test should only be required once. Even if PRSs change over time by adding or removing SNPs from the scoring algorithms as data increases or methodology evolves, individuals with genome-wide data already recorded should be able to take advantage of improvements in PRS accuracy and predictive capacity without giving another sample.

PRS scores also showcase a great opportunity to counsel high genetic-risk patients on lifestyle choices, as noted in the literature [63]. One example would be a patient with a high diabetes PRS. With these results, a physician can discuss with the patient their individual risk, explaining the challenges those with diabetes face, and recommend increasing exercise and a healthy diet. In fact, it may become standard practice to begin hemoglobin A1C monitoring early and to recommend seeing a nutritionist. By counseling a patient prior to disease onset, discussing genetic risk, and starting patients earlier on a healthly lifestyle, the disease can be prevented or caught early enough to reduce disease severity.

One huge advantage of PRSs is that they are far less sensitive to inaccuracies in the genotyping of individual variants than standard clinical genetics procedures. In clinical genetics, we often rely on a single genomic variant for determining a treatment course. Inaccuracies in genotyping can lead to “[failures] to [make] a diagnosis, or [make] a diagnosis in error” leading “to devastating consequences for individuals and families” [2]. For example, reporting absence of common risk variants for breast cancer in single genes such as BRCA1, potentially gives patients a false sense of security, because many other variants may still put the patient at risk. A PRS for breast cancer from a well-powered GWAS meta-analysis could capture a far larger portion of the total genetic risk compared to a single variant.

Simultaneously, individuals on the opposite end of the spectrum will be identified who are at far lower genetic-risk than the population average. Weighing the benefits and risks of screening with such a population will come with its own set of concerns. The decision to delay regular screening or to modify the screening timetable will be a unique decision between patient and physician. As has been discussed in the literature, the recommendation to withhold screening for low- versus high-risk patients will likely be harder to recommend [63], at least until clinicians and health systems become comfortable with the technology and there is a surplus of historical data on the use of PRSs in the clinic upon which to formulate guidelines.

Predictive PRSs also have the potential to identify high-risk groups for medical interventions [7, 63]. One such example is the use of statin therapy in individuals with high cholesterol. Mega et al. found through risk-benefit analysis that those at in the highest-risk category (top quintile) would benefit from therapy initiation. Those who scored in the lower risk categories had less benefit from the medication versus the higher-risk categories [50], leading to the risks (namely of developing diabetes) outweighing the benefits of statin therapy [63]. As information accumulates from the use of electronic medical records and consortia continue to increase in size and diversity, conclusions about who should receive a given therapy and when will become more common. Big data analytics will give healthcare workers the ability to modify their decision-making to reflect the unique genetics of each patient, both increasing benefits and reducing harms on large scales.

Such distinctions and adjustments are not uncommon. Already, clinicians balance the risks and benefits for each patient based on large studies and clinical trials. One such example is the use of calcium channel blockers and thiazide diuretics in African-American patients [6, 69]. These medications are generally preferred to the more standard ACE inhibitors and beta blockers because calcium channel blockers are noted to be more effective and safer to use in this subpopulation [69]. It is not unreasonable for PRSs to expand these discoveries and to expand the efforts of personalized medicine in the future. In fact, the information gathered from genetic data, artificial intelligence, and the electronic medical record combined as big data, is likely to radically change the landscape of medicine on both macro and micro scales.

The COVID-19 pandemic recently demonstrated how important PRSs can also become under exceptional circumstances. Horowitz et al. showed that a high genetic burden from seven common genetic variants that modulate COVID-19 susceptibility and severity was strongly associated with increased risk of hospitalization and severe disease among COVID-19 cases, especially among individuals with few known risk factors. Using genetics to identify individuals at highest risk of adverse outcomes may therefore help prioritize individuals for immunization by (mRNA) vaccines or for treatment with monoclonal antibody treatments when they are still in short supply [27].

## PRS challenges and limitations

### Degree of genetic influence

A fundamental limitation of PRSs is that they typically explain only a small fraction of a trait or disease variance. Unlike monogenic causal gene defects as in the Huntingtin gene or monolithic risk factors like APOE4 haplotype for Alzheimer’s disease, the discriminative ability of PRSs for cancer or CVD is compromised by the environmental factors that come into play, and the imperfect measurement of the full genetic signal that would include structural variants and potentially gene–gene interactions.

However, PRSs correlate with genetic liability, the most prominent single contributor to phenotypic variation. This relationship has allowed for PRSs to be adopted for routine application in biomedical research.

To translate PRSs into clinical tools, relative risks that compare individuals across the PRS continuum with a baseline group will eventually need to be transformed to absolute risks for the disease. For example, scoring very high on a PRS, say the 99th percentile, has almost no meaning if that PRS captures only 1% of variance in disease risk. This is even more true if the prevalence of the disease in the population is low. It is imperative that PRSs are used only in cases were the heritability of a disease is significant and warrants its inclusion. Awareness of this caveat will aid in the prevention of over-reliance on PRSs and acknowledge the need for holistic risk approaches by healthcare professionals and the public.

### Effects of ancestry

Given that allele frequencies and linkage disequilibrium (LD) blocks vary from population to population, especially when these populations are of different ancestries, the genetic profiles of different ancestry groups are significantly different for the purposes of GWAS analysis. In fact, differences in LD blocks can lead to reduced imputation accuracy and incorrect SNP calls for individuals. Additionally, given that PRSs are built using GWAS data, there is an inextricable connection between the genetics and the environment of the GWAS subjects and ultimately the final outcomes of the research [19]. This is especially poignant when sample sizes are small and the environment of the GWAS subjects is fairly similar, leading to reduced capacity to tease out confounding environmental factors within the study population. In turn, these differences result to slightly different effect sizes associated with a given SNP.

While these differences may be small in one instance, given the number of SNPs and the additive nature of current PRS algorithms, the scores generated will be different for those of different genetic ancestries. For this reason, PRSs are essentially locked to the ancestry group (i.e., European ancestry) from which they take their summary statistics and leads to reduced accuracy when applied to individuals of a different ancestry (i.e., Asian ancestry) [19]. Therefore, it is important that an individual’s genetic ancestry be ascertained prior to calculating their PRS. This is often established via principle component analysis to determine the individual’s ancestry followed by imputation based on their associated ancestry group found in a publicly available human genome reference panel such as the 1000 Genomes Project [13].

These issues are increased even more when an individual possess a multi-ancestry background. In these cases, the individual does not fall within either ancestry group and therefore a true score generated for them is unlikely to be a simple averaging of their two or more single-ancestry PRSs. Currently, there is no optimized solution for this segment of the population and more research is needed to develop and test one. These limitations highlight the need for more data from multiple different ancestry groups so that insights from the PRS field can benefit people of all ancestries. Multiple efforts are underway world-wide to address this imbalance which has been discussed by Bentley et al. [5].

### Validation

Currently, the greatest hurdle for PRS implementation in the clinic is the relative lack of validation studies, with very few PRS studies including prospective cohorts to validate the models developed. There are notable exceptions [48] which serve as a model for others to follow, but ultimately this is a systemic problem due to how new PRSs are in the literature. As stated previously, the first large-scale GWAS was only published in 2007 [14], meaning that the knowledge and the underlying infrastructure needed to implement and validate PRSs has only recently been possible. Opportunities for validation for the clinical use of PRSs are expanding through a combination of increasing genome-wide patient data and improved data integration via electronic medical records that are, in turn, using increasingly standardized notation for clinical phenotypes.

Before using PRSs in the clinical setting, they need to go through the same process as any risk factor detected in epidemiological research. PRSs have a distinct advantage over all other risk factors because they can be detected early (i.e., it can signal the need for preventive measures even before onset of clinical signs). In an ideal scenario, PRSs alone should be able to identify the fraction of the overall population that will give rise to the majority of individuals burdened with a given disease. Targeted intervention in these persons will have the highest clinical yield and is the most rational way to spend limited healthcare resources.

The need for validation is two-fold. PRS validation is required to ensure that the patterns seen through the model are accurate to the real world and can therefore accurately predict an individual’s risk of developing a disease [7, 63]. Models can be manipulated to favor certain statistical measures, such as negative and positive predictive value or sensitivity and specificity, but these metrics may be inaccurate if the study does not have a high enough statistical power. In addition, validation of these models is critical to their acceptance by accrediting bodies, insurance companies, hospital systems, clinicians, and patients. The data supporting the accuracy and reliability of these tests must be shown for approval from said groups [63].

**Fig. 3:**
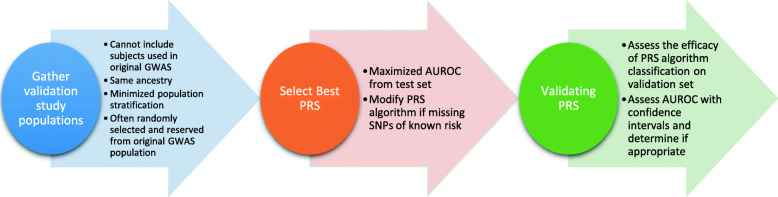
General scheme and important considerations for PRS validation. Note the importance of utilizing a population which was not in the original GWAS used for summary statistic generation but still of identical ancestry composition

Evaluation of PRSs will likely come down to a thorough assessment of the accuracy and reliability of the PRS itself (see Fig. 3), followed by a determination of the clinical efficacy of PRS-tailored prevention or treatment, and finally with an appraisal of cost effectiveness. Assessing the accuracy of PRSs will likely come down to the results of prospective studies and from statistical metrics that determine a high degree of sensitivity for screening, and specificity for alterations of clinical plans for at-risk individuals. Determining the likelihood of an individual to develop a disease, such as breast cancer, emphasizes the need for high sensitivity in order to detect more high-risk patients for other more intensive/aggressive screening methods. On the other hand, modifying treatment regimens, such as altering cholesterol-lowering medications, would likely require a higher degree of specificity so as to not risk exposing patients to adverse side-effects without need [49].

Arguments have been raised about the development of PRSs and how they are validated, especially regarding how PRSs must either be used independently or to be designed in such a way as to be independent of clinical risk factors [33]. Nonetheless, PRS prediction models proved valid in population-based cohort studies and in electronic health record-based studies [20, 44, 45]. While using only independent SNPs would be best in theory, it is less attainable in practice and would minimize the pleotropic nature of human genetics, likely leaving predictive SNPs unaccounted for or further minimize their small effect through approximation as part of associated clinical risk factors. Additionally, this argument assumes that the associated clinical risk factor causes the disease, which may be untrue. That being said, simply adding PRSs to an already established clinical risk algorithm would be unadvised. As stated in Janssens [33], in a combined clinical risk factor and PRS algorithm, both should be optimized for risk prediction [33]. Risk algorithms that combine both genetic and clinical risk factors must be built de novo, validated, and tested prior to use in a clinical setting.

Instead of developing multiple PRSs for the various clinical risk factors associated with a disease (i.e., smoking history and chemical exposure in lung cancer), it would be better to build off of the genetic risk (something inherent and stable) and add clinical risk factors (using weighted factors, etc.) in order to reduce double counting of SNP effects. Ultimately, no model is perfect and the inherent interplay between genetic and environmental factors can never be fully removed, so it is better in this case to embrace and adapt to this reality in the development of a predictive model.

Multiple metrics exist for assessing PRS performance. Area under the receiver operating characteristic (AUROC) curves function well as a composite evaluation of sensitivity and specificity [7, 25] but are less known in the clinical sphere and therefore may require further explanation. Fundamentally, an  AUROC curve is a plot of the sensitivity (or true positive rate) vs. 1-specificity (or false positive rate). The curve denotes this ratio at all possible cut-offs for positive and negative cases and the area under this curve functions as a metric of the PRS’s overall performance across this entire spectrum. Absolute risk (AR), relative risk (RR), and odds ratios (OR) also become vital in accurately quantifying an individual’s risk versus the population to which they are being compared. Other metrics such as negative and positive predictive values (NPV and PPV, respectively) will be useful measures in assessing the benefits of these tools.

Assessing efficacy of PRSs in the clinic is also a challenge. One of the major factors to consider involves how much of a disease’s risk is actually heritable. If the heritability of type II diabetes is only 2%, then a PRS will not be useful because the low level of heritability indicates that there is less of a genetic basis for this disease. Statistically speaking, this is determined as the proportion of variance explained by genetics versus other environmental factors. According to the hypothetical example given above, if genetics, and in turn a PRS, only really explains 2% of an individual’s overall risk for type II diabetes, then a PRS would not be clinically efficacious. Even if an individual’s PRS is in the high-risk category, the absolute risk increase is relatively mild compared to other environmental factors. These scenarios are common when comparing RR and AR for the impact of a given lifestyle or behavior. For these reasons, a proportion of variance and/or the degree of heritability of a given disease should accompany the PRS for clinical purposes.

PRSs beg the question of exactly how useful this knowledge is for treating individuals and in preventing disease overall. In other words, does this knowledge help patients to take charge of their health and to mitigate their risk, or not? We may find, as more and more PRSs are developed, that not all are as effective at adding information to assessing an individual’s risk of disease. For some diseases, the added benefit of genetic information in risk prediction may add very little to the life-time risk of developing the disease, clearly decreasing the efficacy of the PRS. However, the measurement of efficacy regarding clinicians’ awareness of disease risk to better manage, watch for, and intervene should also be considered. This type of information may actually make up for a lack of lifestyle change in a patient and may help clinicians focus on the high-risk diseases in each individual patient.

### Education of clinicians

Clinician education will be a significant challenge as PRSs are implemented into the healthcare system [63]. While knowledge of basic statistics and experimental design is fairly common, especially for younger physicians [37], the specific intricacies regarding GWAS development and PRSs are virtually unknown to clinicians at present [47]. In many ways, this is due to such information being outside of the scope of practice for most physicians and would rarely be needed. However, with the increasing importance of information technology in medicine, the ability to understand and later explain genetic technologies and their nuances to patients will become ever more important.

There are several ways to overcome this challenge. It is common to integrate this type of new tool and information into continuing medical education courses/requirements (also known as CME credits). Through this type of course-work, physicians would be able to work closely with specialists who developed the technology to become familiar with what PRSs are and how they can assist in the clinic for the prevention and management of disease. Importantly, physicians can be exposed to the benefits and detriments of PRSs [7, 26] and to have their questions fielded by experts. CME courses, especially those offered at conferences, also give experts in PRSs the opportunity to workshop with the very physicians they aim to help, to better understand the clinical needs, and to enhance the curriculum to suit the requirements of clinicians.

For medical students, discussions of genetic testing for risk assessment could be phased into regular medical school curricula, likely alongside discussions of relative and absolute risk. The concepts described by PRSs would also lead to important discussions surrounding public health and the importance of regular care, screening, and lifestyle management. Furthermore, activities around the integration of bioinformatics and other informatics tools in the clinic to improve care would be beneficial for physician education.

Another option is to hire genetic risk-assessment specialists who would work closely with physicians to train them on how to interpret the data, implement the information in practice, and coach them on how best to explain the results. These individuals could be integrated into a hospital system or could be hired as outside consultants. Regulations may make consultation for-hire more difficult, but either way would fill the need for training physicians and for serving as a source of information or clarification when questions arise and as the technology evolves.

Alternatively, these same risk-assessment specialists could serve as a new department on their own. Clinicians could refer their patients who have a PRS performed to see this group, similar to how genetic counseling functions in the healthcare system [22]. Perhaps, these risk-assessment specialists would integrate with genetic counselors, working to communicate how the technology works, how the risk score should be interpreted, and how the data should be used for disease prevention. Growing this field to meet the increasing demand may serve as a beneficial resource for healthcare systems, physicians, and patients alike.

### Education of the public

A significant and perhaps more challenging topic surrounding PRSs is how best to communicate the science and, most importantly, the knowledge gained to patients. The concept of risk in general is not difficult to understand, but as mathematics like RR and ORs are introduced the information is quickly lost on the average patient. In general, understanding relative risk is much more challenging than absolute risk. Therefore, it becomes imperative that guidelines for explaining this type of information be developed to ensure that patients are informed but not overwhelmed [23]. Too much information, or too many unnecessary details, may cause patients to forego this type of screening, limiting the ability for PRSs to improve in the future and to identify patients at high risk of disease.

As other papers have stated, it is important to keep PRSs in context [7, 63], discussing the benefits and risks of genetic testing, and emphasizing their likelihood to change as data increases and methods evolve [7, 63]. The idea that a test result might change because we gather more data may be a foreign concept to patients and potentially sew misguided doubts about their validity. Explaining this aspect of PRSs and having a greater conversation about the possibility of changing values will be important topics to discuss.

Limitations of PRSs should also be discussed. Patients come from all walks of life and it is important that clinicians and health systems make a concerted effort to meet patients where they are in their level of knowledge and understanding. The phrase “correlation does not equal causation” is used frequently in science, but one must remember that medicine serves the entire public, with a wide range of socioeconomic, educational, and cultural backgrounds. While this truism may seem obvious to those who read this review, this is not the reality of the clinic.

Genetics as a risk factor for disease is complicated by environmental factors that can play an even larger role. PRSs have been shown to explain only a portion of the total risk of developing a disease, with environmental/lifestyle risk factors making up a much larger proportion [63]. These details are important in helping patients to understand that one’s PRS acts more as a genetic baseline risk while their overall lifetime risk is a combination of genetics and environment. Therefore, it is possible for a patient to overcome a high genetic risk through a healthy lifestyle. Risk scores that integrate both clinical risk factors and a PRS for a given disease will greatly increase the accuracy of lifetime-risk prediction and the intuitiveness of disease risk management. However, it will take time for these tools to be developed and validated for widespread use. For this reason, emphasizing lifestyle choices that can be changed to decrease risk and prevent disease should be the focus when discussing PRSs with patients.

### Healthcare system structures

As more information technologies enter the medical field, new systems must be developed to properly manage and utilize these new tools. This is no different for PRSs, which pose many of the same challenges as standard genetic testing today [15]. The healthcare system must adapt to these changes and develop a system for determining how best to not only integrate PRS into the clinic (as was previously addressed), but also how to implement the technology on a large scale and how to face the fluid nature of PRS-based disease risk assessment.

Assuming the clinical use challenges are settled, healthcare systems still face a major challenge in terms of scaling PRS implementation in the clinic. One major issue that must be tackled before large-scale implementation is cost. More specifically, who bears the cost of the genetic testing required for the process in the beginning. Although genetic testing is becoming more common place in healthcare, running a patient’s genetics for a PRS has not been discussed to date. At the start, PRS assessment will likely fall on individual patients to pay for the test, meaning that the use will be relatively low. Further adoption of this into private insurance and government programs to fund the testing will largely be dependent on considerations such as cost analysis and genomic privacy.

As reimbursement becomes common place, scaling will cause business pressures to increase. While the level of processing power to develop a PRS is not needed to assess an individual patient, computational resources, bioinformaticians, computer programmers, and skilled technicians will still be needed to support the evaluation and troubleshooting of PRSs for patients. In addition, as the number of patients utilizing the service increases, the computational and data storage needs will also increase. This would be a significant cost for each hospital system, especially if they are smaller and have little experience with genetic testing or bioinformatics. Healthcare systems may opt for the use of third parties or other, larger hospital systems to perform this type of analysis, reducing their required initial investment. Such a move may spark the development of new areas for medical information startups increase the utilization of cloud-based storage and processing resources in healthcare.

In addition to scaling, updates and advances to PRSs pose another major problem for hospital systems. As more and more data are collected and methods for analysis are refined, the accuracy and reliability of these scores will improve. Such advances may cause patients to shift from high-risk to intermediate-risk classifications, potentially changing invasive screening and/or treatment recommendations. In addition, if a new update comes out, should all previous patients be rerun against the new algorithm, or only during their annual check-up? Would a hospital system or clinician be held liable for not doing so in a timely manner? The current systems are not designed with such changes in mind and it would be understandable for patients to be confused by and/or upset with changes in standards of care.

Approval and adoption of updated versions become major issues with PRSs. Which body approves a new update? Should the government, such as the U.S. Department of Health and Human Services, or should various medical specialties, like the American College of Cardiology, make such recommendations? How often should these recommendations be updated and who will evaluate the literature? Ultimately, as in the case of most other screening recommendations, the burden of evaluation and recommendation will likely fall under the purview of each country’s specialist society. However, the adoption of these changes may be difficult without a standardized system or program for evaluating the PRS of each patient. This is especially true since not all PRSs will use the same SNPs, nor will all genotyping platforms be the same between healthcare systems. Challenges like this will make the roll out of new versions and the adoption of new standards a complex and chaotic endeavor.

## Improving the PRS field

### Increasing PRS quality

Efforts are already underway to expand biobanking of cellular and genetic materials for use in research. A by-product of these efforts will be an increase in sample sizes for GWASs that are in turn used in the generation of PRSs. With increased sample sizes, disease-associated SNPs are easier to detect and are more likely to reach significance for incorporation in PRSs over time as the field progresses.

Direct measurement, rather than imputation, of SNPs will further improve the predictive capacity of PRSs. While imputation serves as a reliable source of information for genome wide SNP determination, there is still some noise due to using an indirect measurement technique. Direct measurement via more comprehensive array-based genotyping or through next-generation sequencing (NGS) technologies would further increase PRS accuracy and utility, expanding PRS adoption within healthcare systems.

Additional efforts are being made to increase the diversity of populations with available PRSs. Improved diversity is greatly beneficial to ancestry groups that have been overlooked throughout this process due to a lack of sufficient GWAS data available to researchers for the last decade. As of January 2019, 78% of all GWAS data is on individuals of European ancestry [60], severely limiting the potential use and impact of this tool given that non-European populations are growing around the world. Increasing the diversity of GWAS data available for research and development will help to increase the efficacy of PRSs worldwide. This, in turn, may improve currently existing PRSs through a greater understanding of genetic disease architecture [60], by reducing overfitting, and isolating SNPs that are directly causative rather than simply correlated with the disease of interest in a specific ancestry group.

### Enhancing PRS predictive capacity

In practice, at least in the coming years, PRSs will be added as an additional variable to prediction models that use the current set of established risk factors for a particular disease. Models that combine a PRS with established risk factors, including PRS-risk factor interactions, should provide a more precise estimate of the relative risk for the disease in question (cancer, diabetes, CAD, etc.). Given known levels of PRSs and the established risk factors for an individual, the absolute risk over a specified time interval can be computed with this model. Comparing the projected risk to the observed new incidence of disease in a prospective cohort study should then be used to validate the model. Once a good model incorporating a PRS is defined that reliably evaluates absolute risks—that is, the probability that an asymptomatic individual will develop the disease over a certain time interval—it can be used to assign individuals to specific risk categories with differing interventions in an optimized fashion. This means maximizing benefit and minimizing harm associated with unnecessary diagnostic procedures and side-effects of preventative medications and treatments. Already, studies are being published showing that combining standard risk assessment tools with PRSs can improve overall risk prediction [41], even in multiple ancestries [68].

With enhanced capacity to predict the lifetime risk of developing a given disease, more in-depth assessments can be made regarding changes to clinical guidelines surrounding screenings. While suggestions have been made that PRSs could be used to reduce non-invasive interventions for those of lower risk [63], we firmly believe this should only be discussed after PRSs have been fully vetted and sufficient data is available to make such decisions. No model is perfect and there will be individuals with a low PRS for breast cancer, for example, who will still develop the disease, even while mediating all other risks. For this reason, and without double-blinded case-control studies with prospective cohorts, we recommend that PRSs be used exclusively to enhance screening efforts for those at high- and intermediate-risk levels.

Other advances in genomics and sequencing technology will further improve the predictive capacity of PRSs. With the inclusion of other features of genetic architecture (i.e., methylation status, copy number variants (CNVs), structural variants, and sex chromosomes), accuracy, and the level of explained phenotypic variance, will increase. Already, data has shown that the combination of methylation scores with PRSs improves the predictive capacity of various traits, such as BMI and smoking status, indicating the potential for genetic risk modulation via changes in the epigenome (Odintsova et al.: Predicting complex traits and exposures from polygenic scores and blood and buccal DNA methylation profiles, forthcoming). No doubt other genetic factors such as gene silencing and genomic imprinting can also alter the estimation of individual genetic risk. Additional genetic data will help to explain an even greater proportion of the heritability of a given disease. In this regard, a PRS using all SNPs (and potentially all forms of genetic variation) may be used to predict disease risk via an infinitesimal model, acknowledging the complex nature of genetic influence on disease.

### Methods for visualization of polygenic risk

Visualizing the most important result of PRS analyses (the score itself) can aid in transferring this information to both physicians and patients. This also allows for a way to condense the information into a form that is readily interpretable to anyone. There are multiple ways to present PRS information and we will discuss a few examples below. The difficulty ultimately lies in how to balance accuracy, nuance, and clinical applicability. Overwhelming a physician or patient with excessive statistical jargon will lead to PRS results being underutilized if not entirely ignored. Surely, some physicians will jump to utilizing this new technology, but many others will ignore it if too complex and difficult to approach.

**Fig. 4:**
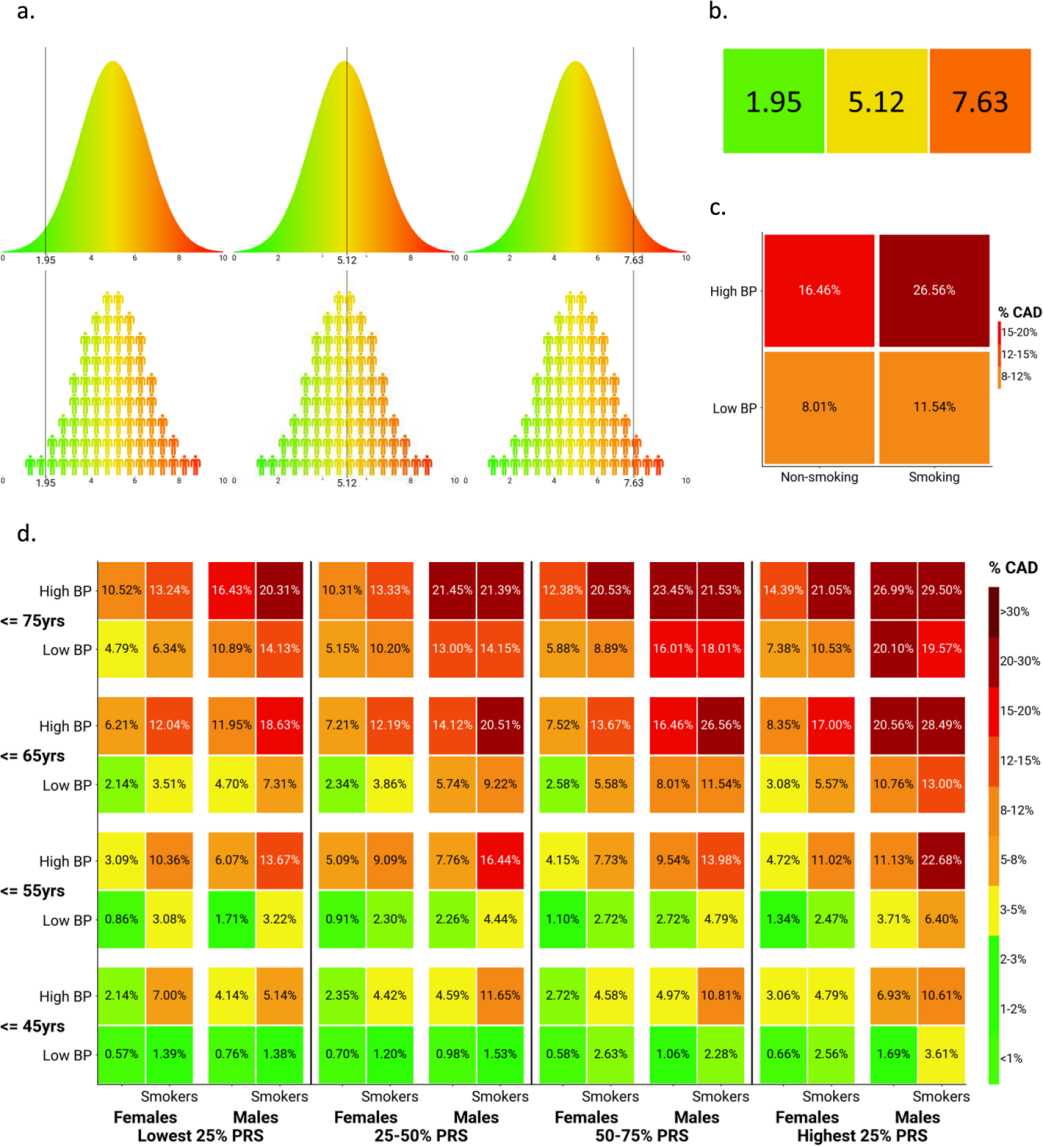
Examples of potential visualization schemes utilizing UK BioBank data for cardiovascular disease (CVD). Polygenic risk scores (PRSs) in **a** and **b** are on a 0–10 scale. **b** Relative risk (RR) can be represented as the scaled score with the colors green, yellow, and red indicating low, average, and high risk, respectively, to help increase understanding. **a** A normal distribution curve can also be used with a scaled score for relative risk along with a green to red gradient representing low to high relative risk of developing CVD within a subject’s lifetime. This graph can be represented with people or a solid color gradient. **d** Clinical factors can also be integrated into a more complex figure modeled from the Framingham Index for CVD with PRS quartiles serving as a static reference for other clinical factors to later adjust the absolute risk (AR) of CVD indicated by the percentage within each square (aka risk block). The AR has been adjusted based on age, sex, smoking history, and blood pressure (BP) in this example. **c** Given known patient factors, a sub-group of risk blocks can be excised from the larger figure for a said patient, showing the improvements to AR by reducing BP and not smoking while still acknowledging the impacts of age, sex, and genetic risk

At its most condensed, the presentation to the physician and/or patient could simply be a number ranging from one to ten, a percentage score, a color, or some combination of these (see Fig. 4b). This method, however, is far from ideal as it expresses relative risk which is less well understood than absolute risk. Translation to absolute risk, however, would depend on the prevalence/incidence of the disease as well as age, sex, and other risk characteristics of the individual. At best, a lot of information and nuance is lost when all data is condensed into a single point. Even worse, if this score, or color, is derived from an underlying normal distribution, the information presented could even be misleading, giving patients a false sense of security about a low risk or a fatalistic view given a high risk due to a lack of contextual information.

To mitigate this slightly, a normal distribution to represent the population can be used (see Fig. 4a for some examples). Note the underlying score (e.g., ranging from 0 to 10) can still be the same, but by including this normal distribution, and making that the main focus of the figure, provides more information and a nuanced understanding. By indicating where the patient falls on this distribution by a line, both patient and physician are able to get a clearer picture of how the patient scored compared to the rest of the population, or even better, compared to a sub-sample of the population which is more comparable to the patient given certain metrics such as age and sex.

While better than a single score, a presentation of the normal distribution may still be flawed. When presented together with other metrics, such as in a diagnostic report, the nuance in relationships and effect sizes is lost. By presenting the PRS score similar to other values such as blood pressure or cholesterol levels, the impression is given that these values are all independent, and of the same effect size. To combat this, a multidimensional plot can be created similar to risk charts that are already commonplace in CVD risk assessment [55]. These plots not only present the information of multiple tests in a single figure, which can reduce the amount of explanation required, but they also inherently include the effect sizes of each phenotype included. The biggest advantage of these plots is that they can include many different phenotypes. Diagnoses or medical advice is rarely, if ever, based on a single measurement, and PRSs should serve as an additional source of information, not a replacement to any one value.

Using data from the UK BioBank [62], we created a binary CVD score. A score of 1 represented any diagnosed incidence indicating CVD, according to ICD-10 diagnosis. And summary statistics from the CARDIoGRAM meta-analysis [58] to compute a PRS for CVD, including sex, age, blood-pressure, and smoking status in addition to the PRS allowed us to generate a new risk chart for PRS (Fig. 4d). From this figure, it is clear that adding the PRS to this chart can be very beneficial. An additional advantage of this layout is that the chart is divided in modifiable phenotypes (blood-pressure and smoking status), and unmodifiable phenotypes (PRS, sex, and age). Therefore, a physician or patient does not need to be presented with the full chart, as the majority does not apply, and will never apply, to the current patient. Only a four-square extract of this plot can be presented. For example, a 60-year-old male patient, who is a current smoker, has high-blood pressure, and who’s PRS falls in the 60th percentile would only be presented a small extract of the chart (Fig. 4c). This not only limits the amount of information presented but would also aid the physician in giving medical advice, e.g., it is clear from this plot that if the patient stops smoking and can lower their blood pressure, their risk of CVD will decrease.

## Ethics of clinical implementation

PRSs have a major potential to revolutionize disease risk-assessment in medicine, but there are multiple ethical considerations to discuss, which are not always intuitive. For instance, it appears to make sense to restrict the use of PRSs to diseases for which effective prevention or early detection strategies are available. However, when such strategies do not exist, Alzheimer’s disease being a current example, the identification of individuals at high risk can still optimize the design of clinical trials to test prevention strategies and/or help in long-term care planning.

**Fig. 5:**
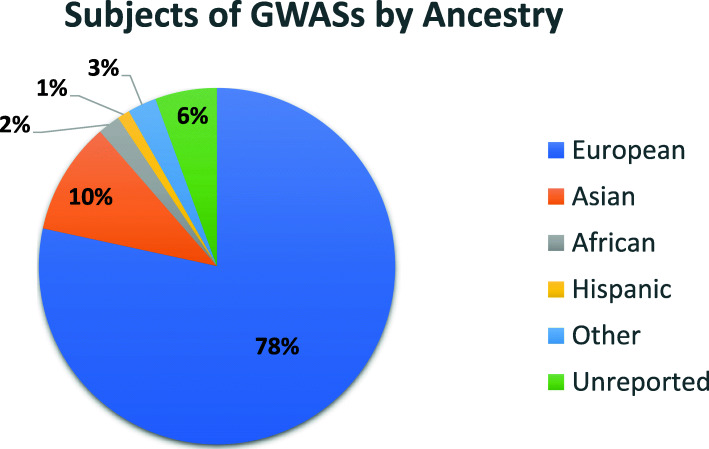
Distribution (%) of total individuals available in the GWAS catalog as of January 2019 [60]

Most ethical concerns derive from the potential mislabeling of persons at high risk as low risk and vice versa. It is therefore crucial to recognize the limitations of a PRS. Firstly, risk stratification based on a PRS alone ignores many other risk factors, including rare monogenic mutations and clinical and environmental factors. Secondly, PRSs are based off of GWAS data from many individuals in a selected population in hopes of representing individuals from the full population. The population you choose to base your PRS off of will narrow the scope of the PRS in question. This is most apparent in that the vast majority of PRSs in the literature are based off of data from individuals with European ancestry (Fig. 5). Other ancestry groups are largely left out of the discussion and the algorithms have not been rigorously tested against these populations.

Therefore, advances in the PRS field have benefited those of specific ancestries, namely those of European descent, with little benefit for other ancestry groups that often suffer higher rates of common diseases [47]. Reasons for this discrepancy include the availability of data in the countries where PRS research is taking place, namely Western nations [60], as well as deeper cultural divisions in medicine (i.e., the distrust of minority patients in the US medical system) [21]. There is a great need for further research and data collection from various ancestries so that the knowledge gained can be shared equitably among all patients. While there are benefits to implementing current PRSs, without such an effort, racial and ethnic health disparities will only widen and further exacerbate the problems faced by patients. It is the moral and ethical imperative of researchers in the PRS field to be aware of this issue and to seek reasonable solutions so that the benefits of this technology are applied universally to all ancestry groups.

Depending on how PRS information is used, it is possible that certain patients may have screening procedures delayed because they fall in a low-risk category. Inevitably, a patient who is determined to be low risk via a PRS will ultimately develop the disease. Will physicians and patients accept this reality? Currently, the system appears to operate only to increase screening as risk factors accumulate for a given patient and those that are low risk stay on the standard schedule for procedures like colonoscopies and mammograms [18, 41, 63]. As risk assessment becomes more quantitatively rigorous, insurance companies may choose to not cover certain screening procedures until a sufficient risk score is obtained for a patient. This is a potentially unacceptable use of PRSs that should be addressed and such changes risk promoting claims of physician and hospital negligence due to denied screening/therapies.

Additionally, reporting PRS information to patients may cause psychological harm to patients and patient apathy [51]. Presented with their genetic risk of a given disease, especially if it is high, some patients may become depressed and fatalistic, choosing to stop all attempts at mitigating risk or rushing to extreme disease mitigation procedures because it is their “genetic destiny” to develop a disease [63]. This use of PRSs is antithetical to the technology’s intention and may exasperate the likelihood of developing the disease in question. While multiple studies have pointed out that the risk is low [12], others have shown more alarming results [64] and we stress the importance of (clinical) guidance in reporting results back to patients. Importantly, a PRS score on its own may be a great disservice to patients, further suggesting the importance of physician and public education. As discussed previously, context is highly important, and the impacts of environmental factors often play a large role in the development of common diseases [29, 30].

## Future directions of PRSs

PRSs, like other bioinformatics technologies, will improve over time as more data is collected and more analytical methods are developed. The number of diseases with associated PRSs is also set to expand via this same endeavor. Already, the National Institutes of Health’s National Human Genome Research Institute has developed a webpage to explain PRSs, how they function, and how they are interpreted.

As the field is still emerging, there is notable heterogeneity in the application and reporting of risk scores, which hinders the translation of PRSs into clinical care. To address this, robust Polygenic Risk Score Reporting Standards (PRS-RS) and a publicly accessible Polygenic Score Catalog have been developed that addresses current research environments with advanced methodological developments to inform clinically meaningful reporting on the development and validation of PRSs in the literature, with an emphasis on reproducibility and transparency throughout the development process [40, 67].

The coming decades will likely see the further expansion of genetic and phenotypic data collection to improve and expand PRSs for multiple ancestry populations and the diseases within those populations. Potentially, ancestry agnostic PRSs will be developed given a sufficient number of subjects from diverse ancestry cohorts. This set of universal PRSs may perform better than ancestry-specific scores because they more closely approach the true genetic risk and reduce the amount of biases, such as overfitting.

Regardless, with increasing data availability for PRS generation, the performance of each algorithm will improve, more diseases will be covered, and more diverse populations will have access to the knowledge PRSs can give. Increased performance will prove to be invaluable in clinical care, especially primary care where many burdensome diseases can be prevented. Since only one blood sample is needed to genotype an individual, genetic risk analysis may be offered to patients at a young age (i.e., early 20s) and enable them to make better health choices for themselves and their loved ones. This bioinformatics-driven change to clinical care may greatly reduce common disease incidence in populations worldwide.

## Conclusion

With our increased understanding of the human genome, the influence of gene-gene and gene-environment interactions, and increasing amount of phenotypic and genetic information available, advances in our understanding of disease and disease risk have continued to expand. Over the last decade, PRSs generated from GWAS summary statistics have been shown to effectively stratify individuals by their life-time genetic risk of developing a given disease. The potential of this new tool is clear and have been noticed by large research bodies such as the NIH in recent years, indicating a significant shift in the use of genetic data in research and medicine.

There are significant benefits that come from PRSs, especially regarding more informed and personalized care. Armed with disease risk information, healthcare professionals can give better advice and recommendations to patients who are at high risk and improve our ability to discover patients who would benefit most from screening and early intervention. From a patient perspective, PRSs offer a single test which can yield a surplus of useful information about potential health risks, empowering them to make healthier lifestyle changes. Some patients may instead take a fatalistic view of PRS information and use it to justify an unhealthy lifestyle, indicating the importance of education and proper communication to prevent this misconception. Disease surveillance would also be improved in the public health domain and could be used to optimize limited funding.

However, PRSs are not without their challenges and risks. Validation will be a major challenge and will require more resources, data, and careful study to ensure the clinical efficacy of PRSs. As a new tool, there are significant barriers to implementation and to understanding how this information should be interpreted. Educating healthcare professionals will be a significant hurdle and reimbursement for testing will likely be slow and require the acceptance of multiple regulatory agencies. While these issues will take time to overcome, they will be as the field grows and advances. Ethical challenges will not be so easily surmounted. The addition of more diverse ancestry populations should be included in discussions about PRS implementation and use. Furthermore, adjusting screening timelines and intervention options should be discussed for the entire disease risk spectrum, but modifications to recommendations for low-risk individuals should only be conducted after thorough evaluation of PRS reliability and accuracy. Such thoughtful changes will help to maintain trust in screening recommendations and ensure that any changes for low-risk individuals maximize benefit while minimizing harm.

PRSs will continue to improve as genetic and phenotypic data increase in size, accuracy, and variety. Efforts to contextualize and explain the nature of PRSs and their uses should become a mainstay of clinical education. Furthermore, regulatory bodies that influence clinical recommendations should prepare for the inevitable introduction of PRSs in normal clinical care, adjusting treatment thresholds and categories around the information this new and exciting tool provide. It is important that clinicians, scientists, regulators, health systems, and patients come together to discuss the benefits and potential risks of PRSs and when and where they should be used.

## Data Availability

Some datasets generated during and/or analyzed within the current work are not publicly available due to the data being privately held by the UK BioBank and accessed via a private data use agreement. Some data are already available in the literature. Other data are available from the corresponding author on reasonable request.

## Abbreviations

AR: Absolute risk
AUROC: Area under the receiver operating characteristic curve
CAD: Coronary artery disease
CME: Continuing Medical Education
CNV: Copy number variant
CVD: Cardiovascular disease
GWAS: Genome-Wide Association Study
LD: Linkage disequilibrium
MI: Myocardial infarction
MRI: Magnetic resonance imaging
NGS: Next-generation sequencing
NPV: Negative predictive value
OR: Odds ratio
PPV: Positive predictive value
PRS: Polygenic risk score
RR: Relative risk
SNP: Single nucleotide polymorphism
